# S-palmitoylation-related genes in Crohn’s disease: Bioinformatic identification and validation

**DOI:** 10.17305/bb.2025.13221

**Published:** 2025-12-09

**Authors:** Yuyan Zhou, Yuxuan Zhao

**Affiliations:** 1 Department of Gastroenterology, Jinan Central Hospital, Jinan, China; 2 Department of Radiology, Qilu Hospital of Shandong University, Jinan, China

**Keywords:** Crohn’s disease, S-palmitoylation, ZDHHC23, IFITM1, immune infiltration, machine learning

## Abstract

Crohn’s disease (CD) is a complex chronic inflammatory bowel disorder characterized by the absence of reliable biomarkers and effective targeted treatments. Recent evidence has suggested a role for S-palmitoylation, a reversible post-translational modification, in immune regulation and intestinal inflammation. However, a systematic, gene-centric investigation explicitly linking S-palmitoylation to the pathogenesis and diagnosis of CD has not been conducted. To address this gap, our study employs a comprehensive bioinformatic analysis to identify and validate key genes associated with both CD and S-palmitoylation, assessing their potential as diagnostic biomarkers and therapeutic targets. Utilizing data from the Gene Expression Omnibus (GEO, GSE83448) and GeneCards, we identified 23 S-palmitoylation-associated differentially expressed genes in CD. Functional enrichment analysis indicated their significant roles in cysteine-specific S-palmitoylation and immunometabolic regulation. We applied machine learning algorithms, including least absolute shrinkage and selection operator regression and support vector machine-recursive feature elimination, to select nine hub genes. Validation in two independent cohorts (GSE16879 and GSE59071) and receiver operating characteristic analysis confirmed *ZDHHC23* and *IFITM1* as biomarkers with high diagnostic value. These genes also exhibited correlations with immune infiltration patterns, as determined by cell-type identification by estimating relative subsets of RNA transcripts, MCPcounter, and QuanTIseq. *In vitro* experiments corroborated consistent changes in mRNA and protein expression for both *ZDHHC23* and *IFITM1*, reinforcing their involvement in CD. This study offers systematic insights into the functional roles of S-palmitoylation-related genes in CD, providing a novel theoretical foundation for the development of diagnostic and targeted therapeutic strategies.

## Introduction

Crohn’s disease (CD) is a chronic and progressive inflammatory bowel disease (IBD) characterized by transmural inflammation that can affect any part of the gastrointestinal tract [1]. The chronic nature of the disease often leads to severe complications, including intestinal strictures, complex fistulas, and perforations, significantly impacting the patient’s quality of life [2]. Currently, the primary treatment approach involves anti-*TNF-α* biologics to alleviate symptoms and slow disease progression [3]. However, approximately 40% of patients either do not respond initially or lose their response over time [4, 5]. Furthermore, long-term immunosuppressive therapy carries risks of infections, cancers, and other autoimmune-related side effects [4, 6], emphasizing the urgent need for new therapeutic targets.

Protein S-palmitoylation is a reversible post-translational modification catalyzed by palmitoyl acyltransferases (PATs), in which palmitic acid or other long-chain fatty acids are covalently attached to cysteine residues [7, 8]. This modification plays a critical role in regulating the membrane localization, stability, and signaling of proteins [9, 10]. It is implicated in various diseases, including cancer progression through the modulation of oncogenic signals, metabolic reprogramming, immune evasion, and the development of neurological dysfunctions [11–15]. Notably, emerging studies have indicated that many molecular pathways related to CD are regulated by S-palmitoylation [16, 17]. For instance, Zhang et al. [18] demonstrated that the NOD-like receptor family, pyrin domain containing 3 (*NLRP3*), undergoes post-translational palmitoylation by *ZDHHC5* at its Leucine-Rich Repeat domain, facilitating the assembly and activation of the NLRP3 inflammasome. Similarly, another study implicated *ZDHHC21*-mediated palmitoylation of Themis, which enhances its protein stability and promotes interactions with *SHP-1* and *Grb2*, leading to the modulation of early T-cell receptor signaling [19].

Despite these compelling associations that highlight the significance of S-palmitoylation in gut homeostasis and inflammation, current evidence remains fragmented. Previous research has primarily focused on individual genes or pathways, and a systematic, unbiased investigation of the entire S-palmitoylation machinery in the specific context of CD is entirely lacking. Additionally, the potential of S-palmitoylation-related genes as diagnostic biomarkers for CD remains unexplored.

Therefore, this study aimed to investigate the dysregulation of S-palmitoylation-related genes in CD and to elucidate their diagnostic potential and immunological roles. To achieve this, we employed integrated bioinformatics approaches to identify and validate key genes, assess their diagnostic value, and determine their association with immune cell infiltration.

## Materials and methods

### Data acquisition and preprocessing

**Figure 1.**
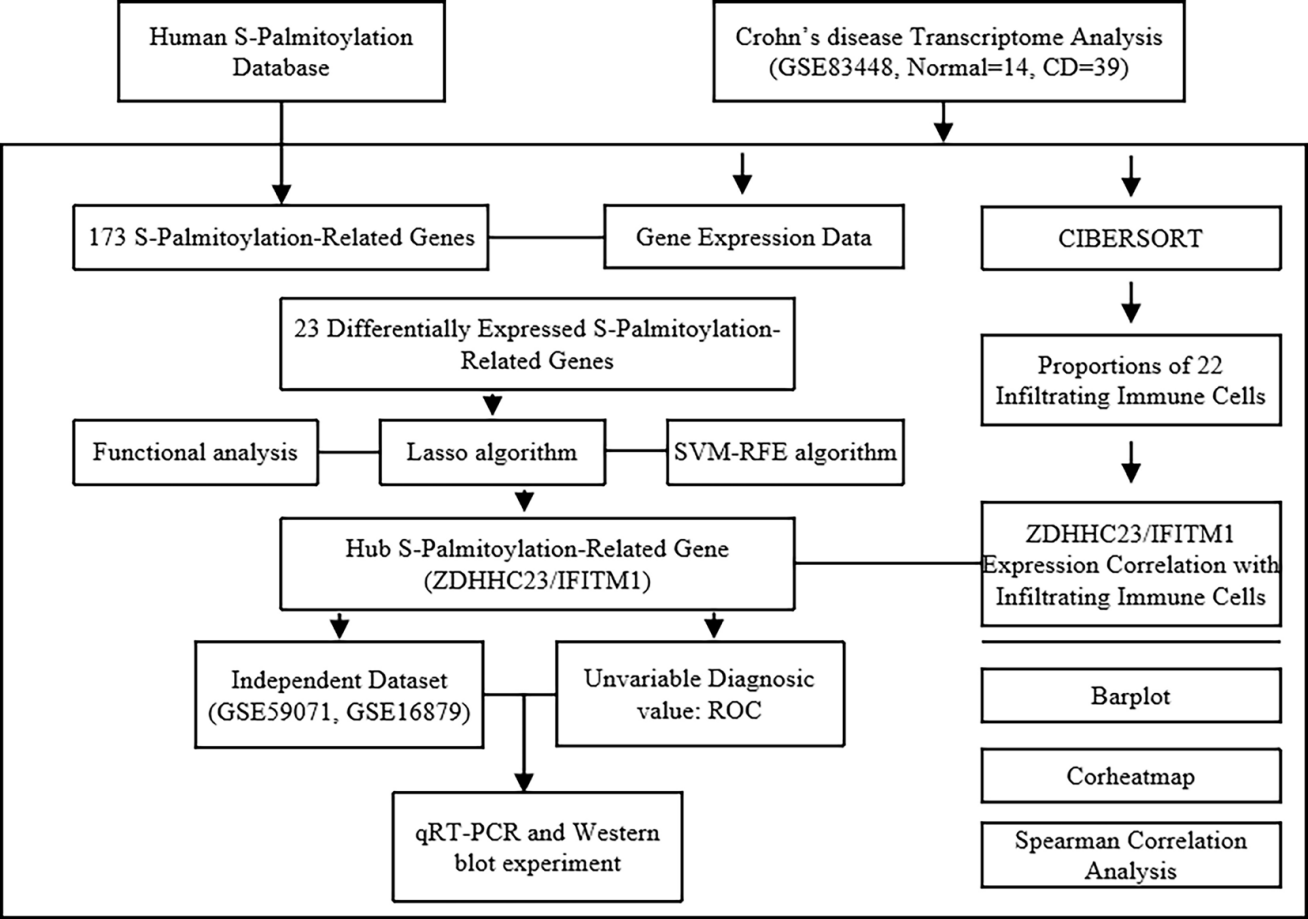
**Flowchart of the study design.** Genomic and transcriptomic data were extracted from the GEO database (GSE83448).

The gene expression microarray dataset GSE83448 was obtained from the National Center for Biotechnology Information Gene Expression Omnibus (NCBI GEO; https://www.ncbi.nlm.nih.gov/geo/) [20]. This discovery cohort comprised gene expression microarray profiles from intestinal mucosal biopsies of 39 CD patients and 14 healthy controls. Two independent validation cohorts (GSE16879 and GSE59071) were additionally retrieved to verify key findings. The workflow is illustrated in Figure 1.

### Identification of S-palmitoylation-related differentially expressed genes (SP-DEGs)

Transcriptomic profiles from the GSE83448 dataset (CD vs healthy intestinal mucosal samples) were subjected to robust normalization and batch effect correction using the “limma” R package [21] (Figure S1). Differentially expressed genes (DEGs) were identified using standard exploratory thresholds of |log_2_ fold change| > 0.3 and an adjusted *P* value < 0.05 (Benjamini–Hochberg false discovery rate [FDR]). Data visualization included volcano plots and principal component analysis (PCA) generated via the “ggplot2” package. S-palmitoylation-related genes were systematically curated from the GeneCards database [22]. Intersection analysis between DEGs and palmitoylation-associated genes yielded SP-DEGs, visualized through Venn diagrams. Expression patterns of SP-DEGs were further illustrated by hierarchical clustering heatmaps and cohort-stratified boxplots.

### Functional enrichment analysis of SP-DEGs

Functional enrichment analysis for SP-DEGs was performed using the Database for Annotation, Visualization, and Integrated Discovery (DAVID) Bioinformatics Database (https://davidbioinformatics.nih.gov/). This analysis encompassed three functional domains: biological process (BP), molecular function (MF), and cellular component (CC) [23]. Enriched terms were filtered by an FDR of <0.05 and visualized using the Xiantao Academic online platform (https://www.xiantaozi.com), which generated graphical representations including hierarchical bubble plots and circular ontology networks [24].

### Machine learning-driven identification of CD hub genes

To optimize the candidate gene library, multi-stage feature selection was conducted on SP-DEGs from the GSE83448 cohort. Initially, least absolute shrinkage and selection operator (LASSO) regression was employed using the “glmnet” package. The optimal regularization parameter (λ) was determined through 10-fold cross-validation, selecting the value of lambda.min that minimized the mean cross-validated error. This L1 penalty term effectively shrank the coefficients of non-informative genes to zero, achieving dimensionality reduction and model parsimony [25, 26]. Concurrently, support vector machine-recursive feature elimination (SVM-RFE) was performed [27]. SVM-RFE utilized a linear kernel with a cost parameter (*C*) of 1, with all features standardized to Z-scores prior to model training. The process was initialized with a random seed of 1234 to ensure reproducibility. SVM-RFE identified 12 key genes from the initial 23 SP-DEGs. The algorithm, facilitated by the “e1071” and “caret” packages, recursively eliminated features and ranked gene importance [28, 29]. The optimal feature subset was identified as the set yielding the highest cross-validated accuracy during the recursive process. To ensure robustness and generalizability, the performance of both models was rigorously validated through a separate, independent 10-fold cross-validation procedure.

The results from these two machine learning approaches were integrated to minimize model error rates, yielding a preliminary core gene set. To further refine this set, the GOSemSim package was employed to conduct Friends analysis, a functional association assessment based on Gene Ontology (GO) semantic similarity [30]. This analysis quantified each gene’s significance within functional networks and generated a ranked list. High-confidence core genes were subsequently screened based on functional importance scores and visualized using raincloud plots.

### Validation of hub gene expression and receiver operating characteristic (ROC) analysis

The differential expression of key genes between CD and healthy intestinal mucosal samples was validated using the GSE16879 and GSE59071 datasets. ROC curves were generated using the pROC package to evaluate the diagnostic value of these hub genes in CD. The area under the curve (AUC) values, along with 95% confidence intervals (CIs), were calculated for each hub gene across all cohorts. Pairwise comparisons of AUCs between cohorts for each gene were performed using DeLong’s test to assess the consistency of diagnostic performance. The optimal cut-off value for each gene in each cohort was determined by maximizing Youden’s J index (Youden’s J = sensitivity + specificity -- 1). The positive predictive value (PPV) and negative predictive value (NPV) were computed based on the observed prevalence of CD within each respective cohort.

### Immune infiltration profiling

The relative proportions of immune cell subsets in intestinal mucosal samples from CD patients and healthy controls (GSE83448 cohort) were quantified using the cell-type identification by estimating relative subsets of RNA transcripts (CIBERSORT), MCP-counter, and QuanTIseq algorithms [31–33]. Spearman’s rank correlation test was employed to assess associations between immune cell infiltration levels and the expression patterns of hub genes. Data visualization, including heatmaps and correlation matrices, was performed using the ggplot2 package in R, revealing significant interactions between S-palmitoylation-associated genes and immunoregulatory cell populations.

### Cell culture

HT-29 cells were obtained from the Cell Bank of the Chinese Academy of Sciences. The cell line was authenticated through short tandem repeat (STR) profiling and tested negative for mycoplasma contamination by the provider. Cells were maintained until reaching 80%–90% confluency before experimental procedures, as described in earlier research [34]. For subsequent experiments, HT-29 cells were stimulated with 1 µg/mL lipopolysaccharide (LPS) for 24 h prior to use. Untreated HT-29 cells served as the control group, providing a baseline for comparison against the LPS-stimulated experimental group. Data from all *in vitro* experiments (quantitative reverse transcription PCR [qRT-PCR] and Western blot) are presented as mean ± SD from three independent biological replicates, with qRT-PCR assays performed in technical duplicate.

### qRT-PCR

Total RNA was isolated from HT-29 cells using the RNAfast200 Total RNA Extraction Kit (Fastagen, Shanghai, China). First-strand cDNA synthesis was conducted via reverse transcription with the SureScript™ First-Strand cDNA Synthesis Kit (GeneCopoeia, Rockville, MD, USA). Quantitative PCR (qPCR) analysis was performed using the LightCycler^®^ 480 Real-Time PCR System (Roche, Basel, Switzerland). All primer sequences are provided in Table S1.

### Western blot

Total proteins were extracted from cells using radioimmunoprecipitation assay (RIPA) buffer (Beyotime, China). Following bicinchoninic acid (BCA) quantification, equal amounts of protein were separated by SDS-PAGE and transferred to polyvinylidene difluoride (PVDF) membranes. Membranes were blocked with 5% bovine serum albumin (BSA) for 1 h at room temperature and subsequently incubated overnight at 4 ^∘^C with primary antibodies diluted as follows: *GAPDH* (1:4000, Proteintech), *ZDHHC23* (1:1000, Epigentek), and *IFITM1* (1:1000, Proteintech). After washing with Tris-buffered saline with Tween 20 (TBST) three times for 10 min each, membranes were incubated with horseradish peroxidase (HRP)-conjugated goat anti-rabbit IgG (1:6000, Proteintech) for 1 h at room temperature. Protein bands were visualized using enhanced chemiluminescence (ECL) and quantified with ImageJ (NIH, USA).

### Statistical analysis

Statistical analyses were conducted using GraphPad Prism 10 and R software (v4.2.0). For comparisons between two groups, the appropriate statistical test (Student’s *t*-test, Welch’s *t*-test, or Mann–Whitney *U* test) was determined based on the results of the Shapiro–Wilk normality test and Levene’s test for homogeneity of variances. A *P* value < 0.05 was considered statistically significant (*P < 0.05,* *P < 0.01, ***P < 0.001, ****P < 0.0001).

For correlation analyses between hub genes and immune cell infiltration (derived from CIBERSORT, MCPcounter, and QuanTIseq), we applied the Benjamini–Hochberg FDR correction to account for multiple testing. Each set of gene–cell pairs within a specific algorithm and cohort was treated as an independent hypothesis family. Associations with an FDR-adjusted *P* value (*q*-value) < 0.05 were considered significant, as indicated by asterisks in the figures.

## Results

### Identification of SP-DEGs in CD

**Figure 2.**
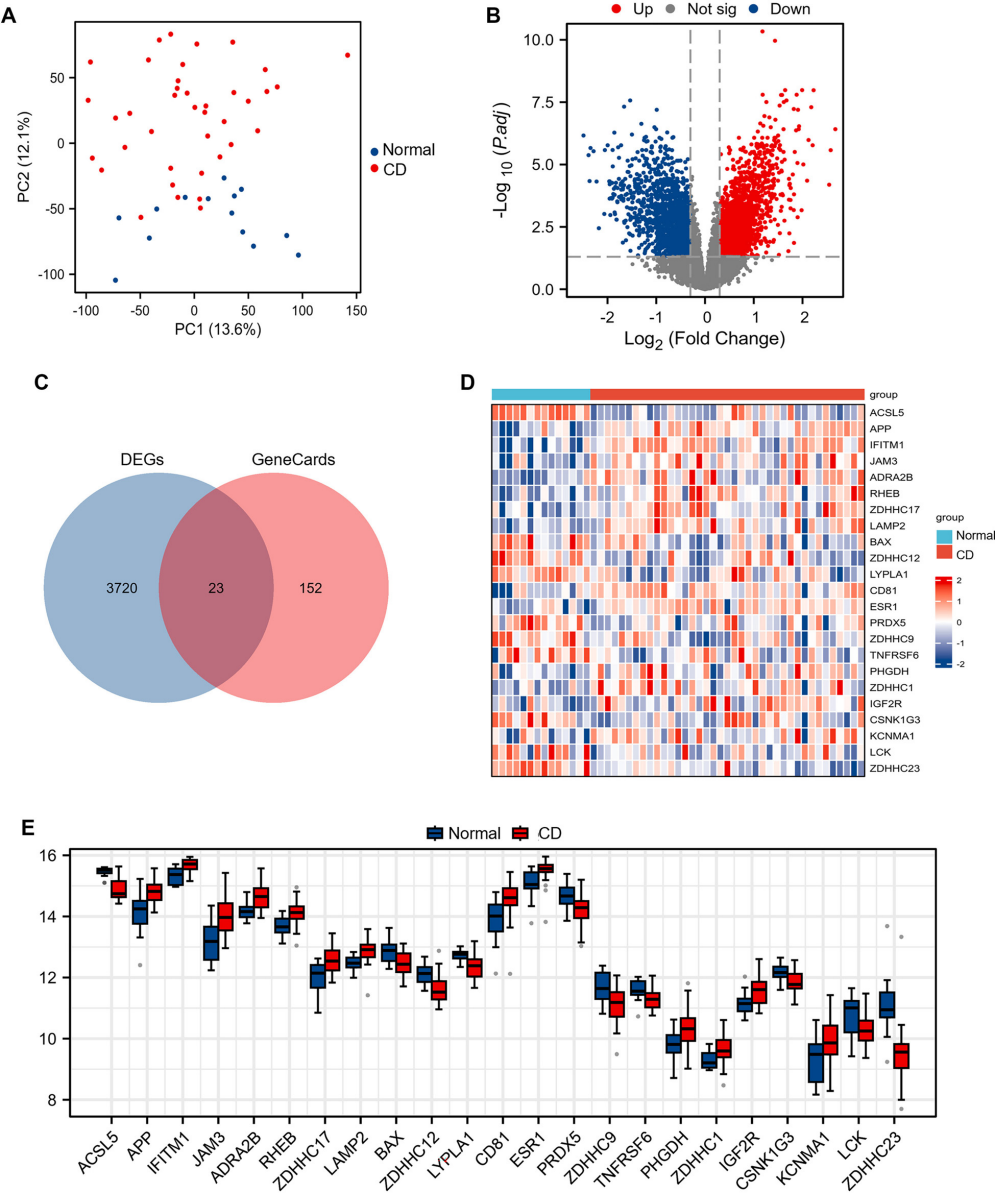
**Differentially expressed S-palmitoylation-related genes in 39 CD intestinal biopsies and 14 healthy controls.** (A) PCA for GSE83448. (B) Volcano plot illustrating 3743 DEGs. (C) Venn diagram depicting the overlap between DEGs and S-palmitoylation-related genes. (D) Heatmap representing the 23 SP-DEGs in CD and healthy controls. (E) Boxplots comparing the 23 SP-DEGs in CD and healthy controls. Abbreviations: CD: Crohn’s disease; PCA: Principal component analysis; DEGs: Differentially expressed genes; SP-DEGs: S-palmitoylation-related differentially expressed genes.

To elucidate the role of S-palmitoylation in CD, we analyzed transcriptomic profiles from the GSE83448 dataset, which includes 39 CD intestinal biopsies and 14 healthy controls. Differential expression analysis identified 3743 DEGs (1992 upregulated, 1751 downregulated; adjusted *P* value < 0.05, |log2FC| > 0.3), with distinct separation between CD and control groups visualized through PCA (Figure 2A) and a volcano plot (Figure 2B).

From the GeneCards database, we curated 173 S-palmitoylation-related genes, including key regulators, such as *ZDHHC* family acyltransferases and acyl-protein thioesterase (*APT*)/palmitoyl-protein thioesterase (*PPT*) thioesterases. Intersection analysis revealed 23 overlapping genes (Figure 2C and Table S2), designated as SP-DEGs. Hierarchical clustering (Figure 2D) demonstrated coherent dysregulation patterns of these SP-DEGs across all samples, while boxplots confirmed significant expression differences between CD and controls (Figure 2E). This systematic approach highlights the potential involvement of dynamic S-palmitoylation in CD pathogenesis.

### Functional enrichment analysis of SP-DEGs

**Figure 3.**
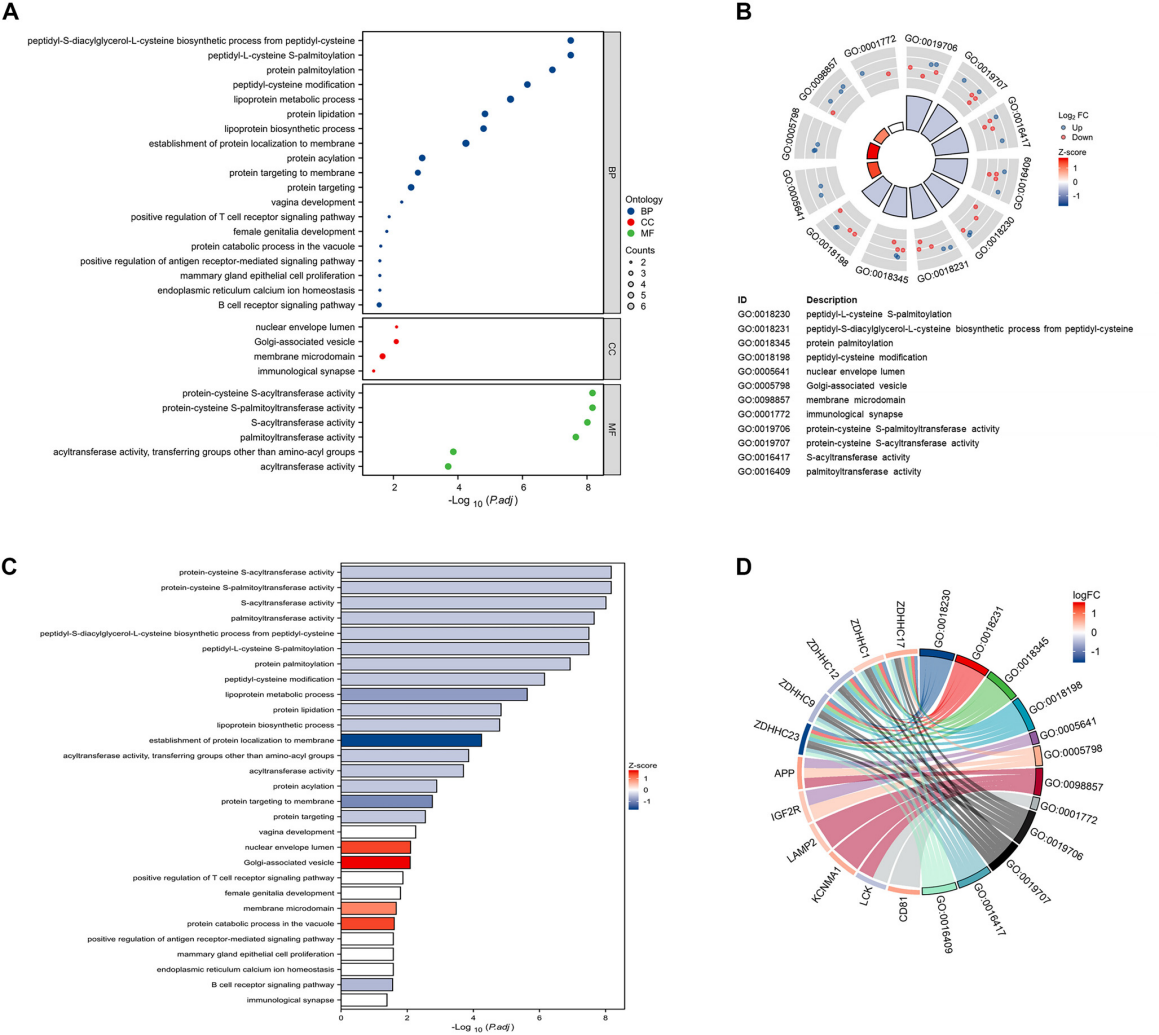
**Functional enrichment analysis of SP-DEGs in the GSE83448 dataset.** (A) The bubble plot illustrates key enriched terms across various categories. BP denotes biological processes; CC indicates cellular components; and MF represents molecular functions. (B) The circle map emphasizes BPs associated with S-palmitoylation. (C) The bar plot presents the results of the functional enrichment analysis, ranked by statistical significance. (D) The Circos plot visualizes the functional associations across categories. Abbreviations: SP-DEGs: S-palmitoylation-related differentially expressed genes; BP: Biological process; CC: Cellular component; MF: Molecular function.

Functional enrichment analysis of the 23 SP-DEGs revealed their predominant involvement in cysteine-specific S-palmitoylation machinery and immune-metabolic dysregulation in CD. BPs were significantly enriched in S-palmitoylation-related terms (GO:0018230) and lipoprotein metabolism (GO:0042157), suggesting a dual role of SP-DEGs in post-translational modification and lipid-mediated inflammatory signaling. MFs emphasized the centrality of S-palmitoyltransferase activity (GO:0019706), with five SP-DEGs encoding key enzymes such as *ZDHHC* family members, which catalyze cysteine palmitoylation of immune receptors and trafficking proteins. CCs localized these genes to critical signaling hubs, including membrane microdomains (GO:0098857) and immunological synapses (GO:0001772), implicating S-palmitoylation in the spatial regulation of pro-inflammatory signaling complexes (Figure 3A–3D). Collectively, these results underscore a cohesive network where dysregulated S-palmitoylation dynamically modulates membrane protein localization, enzymatic activity, and immune cell communication in CD pathogenesis.

### Identification of the S-palmitoylation hub genes in CD

**Figure 4.**
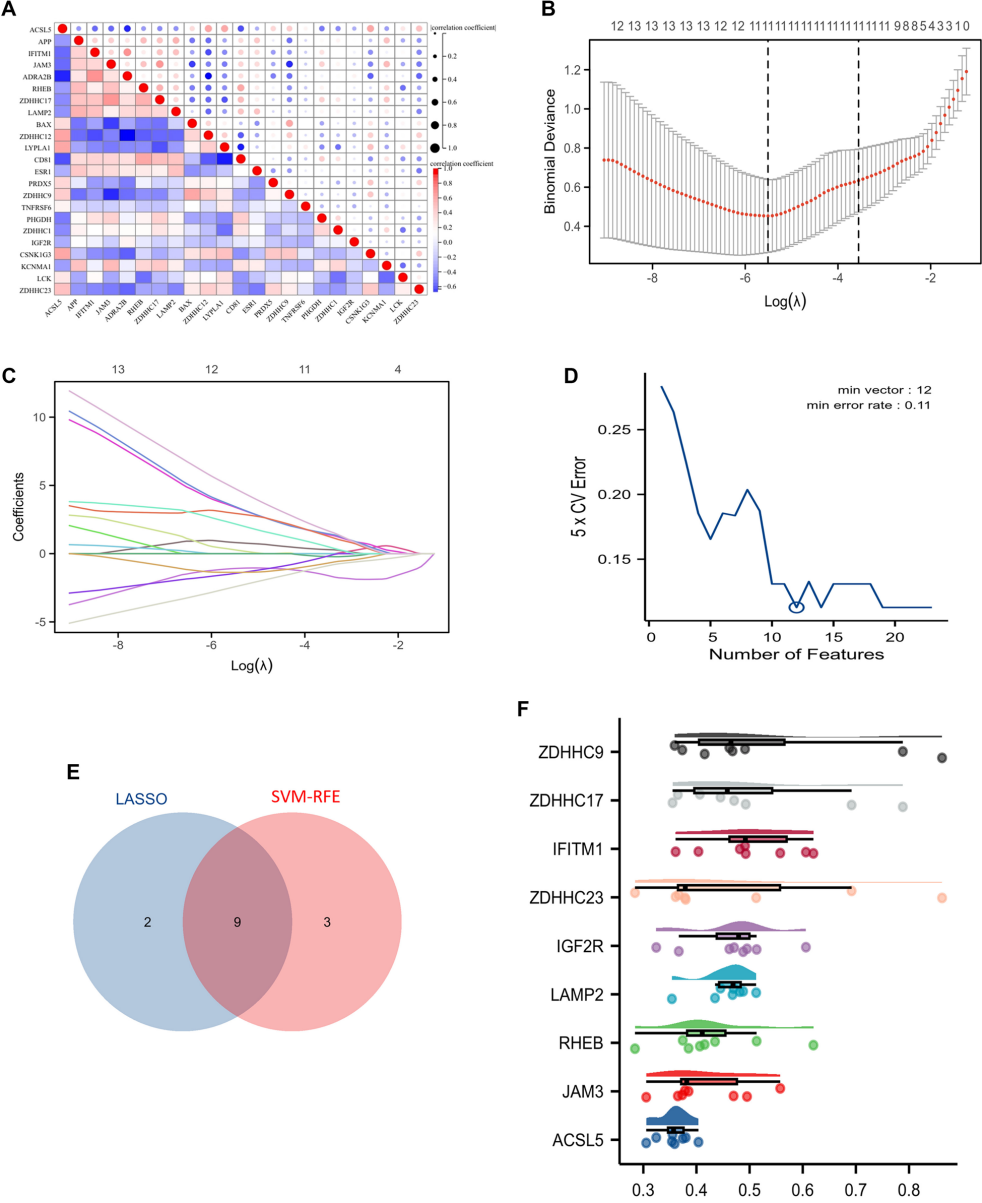
**Identification of S-palmitoylation hub genes in CD.** (A) Spearman’s correlation analysis of the 23 SP-DEGs. (B and C) Eleven candidate biomarker genes were selected from the 23 potential hub genes using the LASSO algorithm. (D) Twelve candidate biomarker genes were identified from the 23 potential hub genes using the SVM-RFE algorithm. (E) The intersection revealed nine genes common to both machine learning algorithms. (F) Raincloud plots illustrate the similarity among the nine genes compared to other genes. Abbreviations: CD: Crohn’s disease; SP-DEGs: S-palmitoylation-related differentially expressed genes; LASSO: Least absolute shrinkage and selection operator; SVM-RFE: Support vector machine-recursive feature elimination.

To investigate the coordinated regulation among the SP-DEGs, we first performed co-expression network analysis, revealing tightly clustered interaction modules (Figure 4A). Subsequently, dual feature selection was conducted using LASSO regression and SVM-RFE algorithms. LASSO regression employed L1 regularization to minimize coefficients of non-critical variables, identifying 11 candidate core genes for CD (Figure 4B and 4C). Concurrently, the SVM-RFE algorithm selected 12 candidate core genes through iterative feature weight optimization (Figure 4D). Venn diagram analysis revealed nine consensus candidate core genes shared by both algorithms: *ZDHHC9, ZDHHC17, IFITM1, ZDHHC23, IGF2R, LAMP2, RHEB, JAM3*, and *ACSL5* (Figure 4E). Finally, Friends analysis prioritized these genes by importance ranking, leading to subsequent experimental validation (Figure 4F).

### Multi-cohort validation of S-palmitoylation hub gene in CD

**Figure 5.**
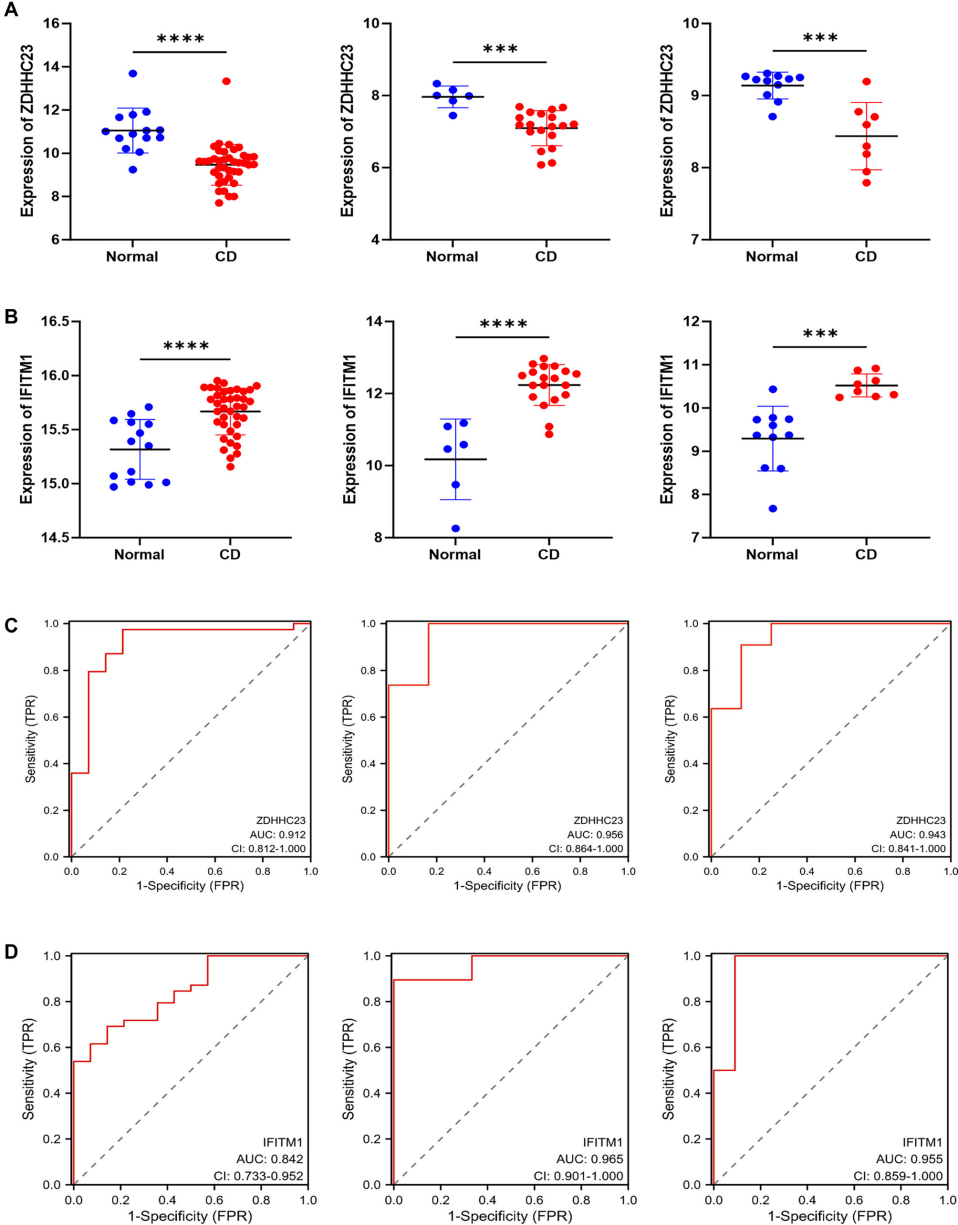
**Multi-cohort validation of *ZDHHC23* and *IFITM1* as diagnostic biomarkers for CD.** (A) Significant downregulation of *ZDHHC23* expression across multiple CD cohorts. (B) Significant upregulation of *IFITM1* expression across multiple CD cohorts. (C) ROC curve illustrating the diagnostic utility of *ZDHHC23* in CD detection. (D) ROC curve illustrating the diagnostic utility of *IFITM1* in CD detection. Asterisks indicate statistical significance (***P < 0.001; ****P < 0.0001). Abbreviations: CD: Crohn’s disease; ROC: Receiver operating characteristic.

The expression patterns of nine hub genes were initially examined in the discovery cohort (GSE83448) and subsequently validated in two independent external cohorts (GSE16879 and GSE59071). This analysis revealed a significant downregulation of *ZDHHC23* (*P* < 0.001) and a marked upregulation of *IFITM1* (*P* < 0.001) in CD patients compared to controls (Figure 5A and 5B, Figure S2, and Table S3). ROC analysis demonstrated the excellent diagnostic capacity of the hub genes for CD. Specifically, *ZDHHC23* achieved an AUC of 0.956 (95% CI: 0.864–1.000) in the GSE16879 validation cohort. Overall, all hub genes maintained AUCs above 0.80 across independent cohorts (Figure 5C and 5D and Table S4). Pairwise comparisons using DeLong’s test revealed no statistically significant differences in AUC values between cohorts for any hub gene (all *P* > 0.05, Table S5), supporting consistent diagnostic performance across diverse patient populations.

### Analysis of immune cell infiltration

**Figure 6.**
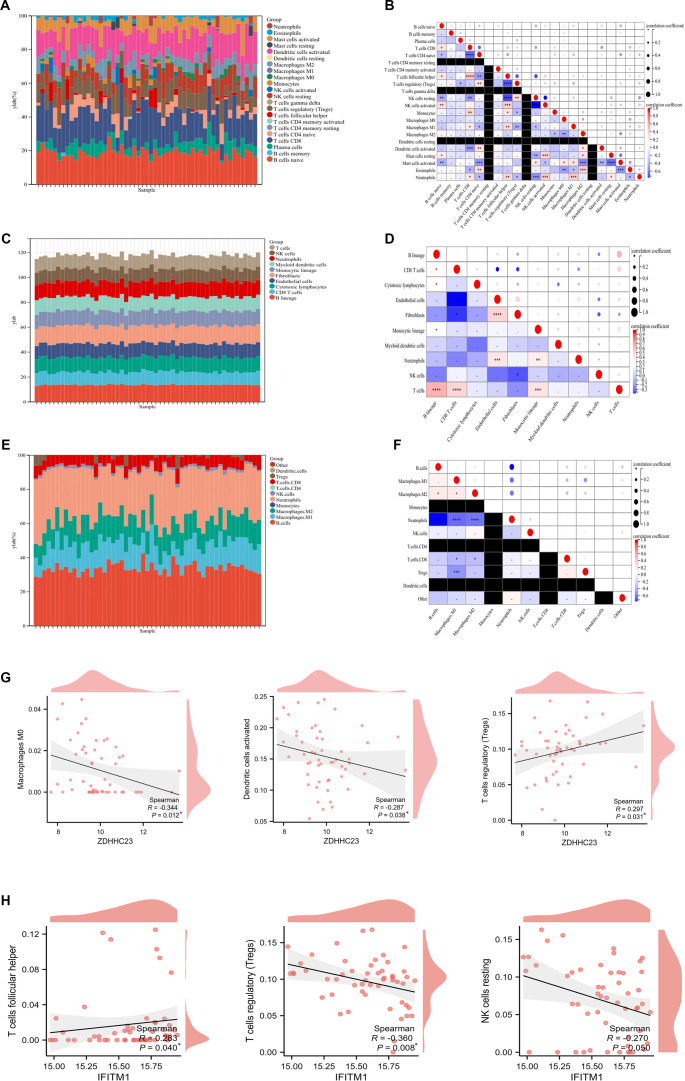
**Visualization of immune cell infiltration.** (A and B) The correlation matrix of infiltrating immune cell composition and proportions was generated using the CIBERSORT algorithm. (C and D) The correlation matrix of infiltrating immune cell composition and proportions was produced using the MCPcounter algorithm. (E and F) The correlation matrix of infiltrating immune cell composition and proportions was established using the QuanTIseq algorithm. (G) Correlation between *ZDHHC23* expression and infiltrating immune cells, specifically M0 Macrophages, activated dendritic cells, and regulatory T cells. (H) Correlation between *IFITM1* expression and infiltrating immune cells, including follicular helper T cells, regulatory T cells, and resting NK cells. Asterisks (*) indicate correlations that remained statistically significant after FDR correction for multiple testing across all gene–cell pairs within each algorithm (*q* < 0.05). Abbreviations: CIBERSORT: Cell-type identification by estimating relative subsets of RNA transcripts; FDR: False discovery rate; NK: Natural killer.

Using three deconvolution algorithms (CIBERSORT, MCP-counter, and QuanTIseq), we conducted an immune infiltration analysis on the GSE83448 dataset. To account for false positives due to multiple comparisons, all correlation analyses underwent Benjamini–Hochberg FDR correction, with a *q*-value < 0.05 considered statistically significant. Correlation matrix analysis identified the strongest negative interaction between resting natural killer (NK) cells and activated NK cells (Figure 6A–6F).

After FDR correction, *ZDHHC23* expression maintained a significant positive correlation with regulatory T cells (Tregs) and significant negative correlations with pro-inflammatory M0 macrophages and activated dendritic cells (*q* < 0.05; Figure 6G). Conversely, for *IFITM1*, only its positive correlation with T follicular helper cells and negative correlation with Tregs remained significant after FDR correction (*q* < 0.05; Figure 6H and Table S6). The correlation landscape for *ZDHHC23* across a broader panel of 12 immune subsets is detailed in Figure S3, indicating FDR-significant associations. A similar comprehensive analysis for *IFITM1* (Figure S4) revealed that, after FDR correction, its correlations with other immune cell types (including naive B cells, CD8^+^ T cells, and M0 macrophages) were not statistically significant (all *q* ≥ 0.05). These findings imply that *ZDHHC23* and *IFITM1* may constitute a counter-regulatory axis in CD, where lower *ZDHHC23* levels are associated with reduced immune tolerance, while higher *IFITM1* levels correlate with amplified effector T-cell responses.

### Biological validation of hub genes

**Figure 7.**
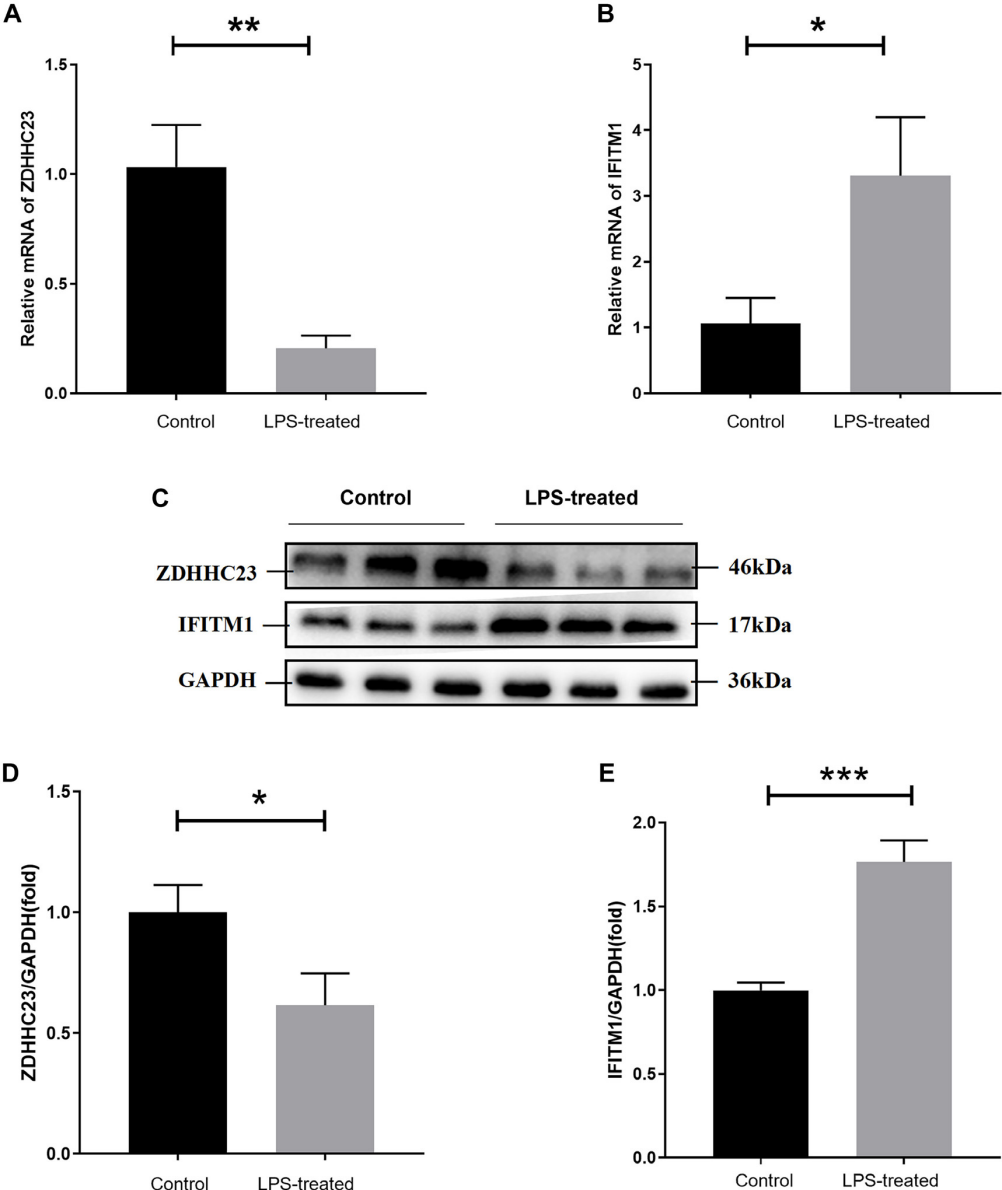
**Experimental validation of *ZDHHC23* and *IFITM1* expression in LPS-treated HT-29 cells.** The expression levels of *ZDHHC23* and *IFITM1* in HT-29 cells were validated through qRT-PCR analysis, which confirmed mRNA levels for *ZDHHC23* (A) and *IFITM1* (B). Western blot analysis (C) demonstrated protein expression differences between control and LPS-treated HT-29 cells (*n* ═ 3 biological replicates). Densitometric quantification of protein expression was performed for ZDHHC23 (D) and IFITM1 (E). Asterisks indicate statistical significance (*P < 0.05; **P < 0.01; ***P < 0.001). Abbreviations: LPS: Lipopolysaccharide; qRT-PCR: Quantitative reverse transcription PCR.

To further validate our findings, we established an *in vitro* model of CD using LPS-stimulated HT-29 cells. qRT-PCR and Western blot analyses were conducted to assess expression changes in the key genes *ZDHHC23* and *IFITM1*. Results indicated significantly reduced mRNA expression levels of *ZDHHC23* and markedly elevated expression of *IFITM1* in LPS-treated groups compared to controls (Figure 7A and 7B). Western blot analysis corroborated these findings, revealing corresponding trends of downregulation in ZDHHC23 protein and upregulation in IFITM1 protein (Figure 7C–7E). These observations align with our prior analytical conclusions and suggest that the downregulation of ZDHHC23 and upregulation of IFITM1 may contribute to the pathogenesis of CD.

## Discussion

This study integrates bioinformatics and machine learning approaches to elucidate the role of S-palmitoylation in CD, identifying *ZDHHC23* and *IFITM1* as central regulatory hubs that connect post-translational modifications to immune-metabolic dysregulation. Both biomarkers exhibited consistent diagnostic performance across multiple independent cohorts, with their clinical potential bolstered by a comprehensive ROC analysis encompassing sensitivity, specificity, PPV, and NPV. Our findings not only enhance the understanding of CD pathogenesis but also propose novel therapeutic targets for this challenging condition.

The identification of 23 SP-DEGs underscores the critical role of dynamic lipid modifications in CD. Functional enrichment analysis indicates that these genes are primarily involved in immune synapse organization (GO:0001772) and lipoprotein metabolism (GO:0042157), which align with the dual pathological features of CD: chronic inflammation and metabolic reprogramming [35, 36]. Notably, the downregulation of *ZDHHC23* implies a disruption in the palmitoylation of immune receptors or barrier integrity proteins. This is consistent with prior evidence that impaired S-palmitoylation exacerbates mucosal inflammation by destabilizing tight junctions and enhancing nuclear factor kappa B (NF-κB) signaling [37–39].

The strong negative correlation between *ZDHHC23* and M0 macrophages further implicates its role in suppressing pro-inflammatory myeloid activation. Mechanistically, *ZDHHC23* may influence macrophage polarization by palmitoylating RAC1 or STAT3, both of which are critical for maintaining the M1/M2 balance [40–42]. While our study identifies *ZDHHC23* as a promising therapeutic target, we emphasize the necessity of functional validation through knockout models and compound screening prior to clinical translation. Conversely, its positive association with regulatory T cells (Tregs) suggests a protective role in sustaining immune tolerance, potentially through palmitoylation-dependent stabilization of FOXP3 or CTLA-4 [43–45]. These findings position S-palmitoylation as a rheostat that fine-tunes immune homeostasis in the gut. The consistent downregulation of *ZDHHC23* across discovery and validation cohorts (GSE83448 and GSE16879) and its high diagnostic accuracy (AUC > 0.85) underscore its clinical relevance. This parallels studies in cancer, where the loss of *ZDHHC23* drives metastasis through RAS depalmitoylation [46, 47], suggesting a conserved mechanism across inflammatory and neoplastic contexts. Notably, the dysregulation of *ZDHHC23* may account for the secondary failure of anti-TNF therapies in CD, as tumor necrosis factor-alpha (TNF-α) signaling requires the palmitoylation of TNFR1 for proper membrane localization [48]; decreased *ZDHHC23* activity could impair TNFR1 trafficking, reducing drug efficacy [49, 50]. Targeting *ZDHHC23* or its substrates may restore therapeutic responsiveness, providing a precision medicine approach for non-responders.

Furthermore, this study elucidates the dual role of *IFITM1* in CD, where its consistently elevated expression in CD patients and LPS-stimulated intestinal epithelial cells correlates positively with follicular helper T cells (Tfh). These findings suggest that *IFITM1* may enhance mucosal antibody responses by promoting Tfh-mediated B-cell activation, consistent with previous reports of *IFITM1* facilitating inflammasome activation in inflammatory contexts [51, 52]. The stable high AUC values across validation cohorts reflect IFITM1’s sensitivity to intestinal inflammation, likely due to its classification as an interferon-stimulated gene (ISG) that undergoes synergistic induction by TNF-α and interferon-gamma (IFN-γ) within inflammatory microenvironments [53, 54]. Importantly, *IFITM1* is a confirmed palmitoylation substrate (Cys72/83/105), and its functional dynamics are dependent on modification status. Palmitoylation enhances IFITM1’s localization to lipid rafts, facilitating its inhibition of viral membrane fusion [54]. In CD, while we observed increased IFITM1 protein levels, its palmitoylation efficiency requires further validation. Collectively, our results indicate that IFITM1’s role in CD extends beyond conventional interferon effector functions.

Several limitations should be acknowledged when interpreting our results. First, the use of non-nested cross-validation in our machine learning feature selection may introduce optimistic bias in performance estimates. Although we employed independent validation cohorts to mitigate this concern, future studies would benefit from nested cross-validation frameworks for more robust performance metrics. Second, the association between *ZDHHC23*/*IFITM1* and immune dysregulation remains correlative; *in vitro* experiments, such as CRISPR-Cas9 knockdown in intestinal organoids, are necessary to validate the functional impact on barrier integrity and cytokine production. Third, bulk RNA-seq data from mucosal biopsies may obscure cell-type-specific palmitoylation dynamics, and single-cell sequencing could address spatial heterogeneity. Additionally, while our *in vitro* experiments utilized a sample size of *n* ═ 3 biological replicates, which is standard in the field, we acknowledge that this modest sample size warrants consideration. However, the consistency of our findings across both mRNA and protein levels, coupled with the significant effect sizes observed, lends confidence to the robustness of these results. Lastly, while PAT inhibition has been explored in cancer [55, 56], therapeutic translation for CD requires complementary approaches: developing *ZDHHC23*-targeted agonists to restore anti-inflammatory palmitoylation alongside IFITM1-neutralizing biologics to disrupt its membrane-associated pro-inflammatory pathways. Moreover, the strong diagnostic performance of both *ZDHHC23* and *IFITM1* supports their potential as clinical biomarkers for CD stratification and monitoring. Future work should address the challenges of developing specific palmitoylation-targeted therapies and validating these biomarkers in larger clinical cohorts to establish their definitive diagnostic and therapeutic utility.

## Conclusion

This study systematically identifies SP-DEGs in CD through comprehensive bioinformatics analysis. Among these, *ZDHHC23* and *IFITM1* were established as hub biomarkers demonstrating consistent diagnostic value across multiple validation cohorts. Experimental validation confirmed their dysregulation at both transcriptional and protein levels, while immune infiltration analysis revealed significant correlations with specific immune cell subsets. These findings not only provide novel insights into the molecular mechanisms of S-palmitoylation in CD pathogenesis but also offer potential biomarkers for diagnostic development and therapeutic targeting.

## Supplemental data

Supplemental data are available at the following link: https://www.bjbms.org/ojs/index.php/bjbms/article/view/13221/4077.

## Data Availability

The data that support the findings of this study are available in Gene Expression Omnibus (datasets with identifiers: GSE83448, GSE16879, and GSE59071), which were downloaded from http://www.ncbi.nlm.nih.gov/geo/.
